# Citizens Attitudes about the Emergency Situations Caused by Epidemics in Serbia

**Published:** 2018-08

**Authors:** Vladimir M. CVETKOVIĆ, Elizabeta RISTANOVIĆ, Jasmina GAČIĆ

**Affiliations:** 1. Faculty of Security Studies, University of Belgrade, Gospodara Vucica 50, 11000 Belgrade, Serbia; 2. Military Medical Academy, University of Defence in Belgrade, Belgrade, Serbia

## Dear Editor-in-Cheif

Epidemics increasingly threaten the safety and health of people. As such, epidemics more and more begin to attract the attention of researchers in the field of emergencies who want to know them better. Natural disasters contribute to the spread of much serious food and water-borne diseases, primarily due to compromised or disrupted water and sewage systems (1). Poor hygiene can be a huge challenge immediately after a natural disaster, especially if the victims are displaced and/or find refuge in shelters (2). “Infectious diseases were detected in 85% of all patients, predominantly malaria, respiratory infectious diseases, and diarrhea” (3). The twentieth century in Europe remains remembered for the Spanish flu pandemic which caused more victims than World War I, while Serbia had the great epidemic of typhus during World War I in 1915 and the epidemic of smallpox in 1972, recorded as the largest post-war epidemic in Europe. Today, smallpox again represents a threat due to the vulnerability of the population (4).

Starting from the impact of the epidemics on humans, the authors present the results of a quantitative survey of citizens’ attitudes and influencing factors on emergencies caused by epidemics in Serbia. Thereby, the survey focused on the examination of knowledge of citizens about epidemics and the proper ways to respond; citizens’ preparedness for epidemics; and having a first aid kit. A series of 2500 face-to-face interviews were conducted during the whole of 2015 in 19 municipalities of Serbia. Verbal informed consent was taken from the participants before the study. These communities were chosen for their different demographic and social characteristics being a census-based representation of the whole population of Serbia.

In the past, the right to education in emergency situations was not affirmed and recognized (5). Given the importance of citizens’ knowledge about epidemics for improvement of their response in emergencies, respondents were asked the following questions: “Do you know what epidemics are and how to protect yourself against them? Of those surveyed, 43.1% of the respondents know what epidemics are and how to protect themselves against them, 26.6% were not sure and 24.7% did not know. Less than half of respondents know what epidemics are and how to protect themselves against epidemics. The results indicate a serious security problem, considering the knowledge as a prerequisite for an effective response in such situations. The results of Chi-square test of independence showed that there is a statistically significant correlation between knowledge about epidemics and how to protect against them with the following variables: gender, age, level of education, level of mother education, level of father education, marital status, parental status, fear of disaster, personal disability, employment status, income level of households, level of religiousness, previous experience, volunteering, military status regulated. On the other side, there is no such correlation with variable called living with a disability. There is a statistically significant correlation with all mentioned variables except volunteering and military status regulated. In higher percentage know what epidemics are and proper way to respond: women (47.8%), with university degrees (54%), who have finished high school with honors (59%), married (53.6%), who are parents (52%), who are not disabled (47%), employed (50.7%), who feel fear (50%), with previous experience (61.1%).

Consequences of disaster are impossible to avoid. However, an adequate system of management can mitigate them. Preparation of citizens for disaster is influenced by a variety of social and individual factors. Thereby, these directly or indirectly affect the citizens to implement, take or develop preparedness measures for responding in such situations. Understanding their influence is an important step towards devising ways of raising the level of citizens’ preparedness. The mean of citizen preparedness for responding is 2.98 out of 5. Descriptive statistical analyses showed that 28.2% of respondents were unprepared to respond, 44% were not sure and 26.6% that they are prepared. A very small number of respondents said that they are prepared for responding to emergencies caused by epidemics. In higher percentage prepared respondents are: men (32.4%), with university degrees (29.4%), have completed high school with very good grades (27.2%), in relationships (32.7%), not parents (32%), take care of a disabled person (34%), without disabilities (26.7%), who feel fear (29%).

The citizens also were asked if they have a first aid kit. Of all respondents, 47% have a first aid kit at home, 37.8% were not sure and 25.2% do not have. There is a statistically significant correlation with all mentioned variables except gender, level of father education, personal disability, and previous experience. In a higher percentage a first aid kit has respondents: university-educated (55.4%), finished secondary school with honors (56%), divorced (53.7%), parents (54%), employed (52%), respondents who feel fear (55%).

Starting from the unexamined citizens’ attitudes toward epidemics in Serbia, the research has original scientific and social significance. Knowledge of citizens can be improved through educational television or radio shows which would inform citizens about possible epidemics. Starting from the presented research results, it is necessary to devise campaigns and programs aimed at improving the citizen’s knowledge and preparedness for epidemics.
